# Tandem Duplication of Serpin Genes Yields Functional Variation and Snake Venom Inhibitors

**DOI:** 10.1093/molbev/msaf290

**Published:** 2025-11-11

**Authors:** Meilyn S Ward, Matthew L Holding, Laura M Haynes, Mark J Margres, Marjorie D Matocq, David Ginsburg

**Affiliations:** Life Sciences Institute, University of Michigan, Ann Arbor, MI, USA; Life Sciences Institute, University of Michigan, Ann Arbor, MI, USA; Department of Ecology and Evolutionary Biology, University of Michigan, Ann Arbor, MI, USA; Life Sciences Institute, University of Michigan, Ann Arbor, MI, USA; Department of Integrative Biology, University of South Florida, Tampa, FL, USA; Department of Natural Resources & Environmental Science; Program in Ecology, Evolution, and Conservation Biology, University of Nevada Reno, Reno, NV, USA; Life Sciences Institute, University of Michigan, Ann Arbor, MI, USA; Department of Internal Medicine, University of Michigan, Ann Arbor, MI, USA; Department of Human Genetics, University of Michigan, Ann Arbor, MI, USA; Department of Pediatrics, University of Michigan, Ann Arbor, MI, USA

**Keywords:** neofunctionalization, serine protease, resistance, coevolution, predator-prey, rodent

## Abstract

Tandem duplication of genes can play a critical role in the evolution of functional novelty, yet our understanding is limited concerning the role of gene duplication in coevolution between species. Much is known about the evolution and function of tandemly duplicated snake venom genes, however, the potential of gene duplication to fuel venom resistance within prey species is poorly understood. The SERPINA subfamily of genes produces globular serine protease inhibitors and carrier molecules, and SERPINA1 has previously been shown to inhibit snake venom serine proteases. In this study, we characterize patterns of duplication within the tandem array of SERPINA, documenting trends in copy number evolution between species. We find the hallmarks of rapid birth-death evolution of SERPINA1-like and SERPINA3-like genes within and between rodent lineages, and evidence for diversifying selection acting on rodent genes. To explore the functional significance of copy number evolution, we recombinantly expressed the full set of 12 paralogous duplicates of SERPINA3 found in the genome of the Big-eared woodrat (*Neotoma macrotis*), a species known for resistance to protease-rich rattlesnake venoms. Two SERPINA3 paralogs inhibited venom serine proteases, indicating that these proteins may serve as resistance factors. In addition, functional variation is apparent among paralogs, including neofunctionalization to inhibit both chymotrypsin-like and trypsin-like proteases simultaneously for one venom-inhibiting paralog. Our results provide further evidence that the rapid evolution of SERPINA1 and SERPINA3 gene copy number across rodents has adaptive potential by producing functionally diverse venom inhibitors.

## Introduction

Coevolution governs many interactions in nature, from mutualism to parasitism and predator-prey relationships (Brodie et al. 2005; Thompson 2005; Wu et al. 2023 ). The escalating “arms race” is a common metaphor for the latter case, especially, when describing pairs of morphological, physiological, or molecular traits that evolve in response to changes in the coevolving species (Brodie et al. 2002; Benkman et al. 2003; Toju 2008). One potential evolutionary response to reciprocal selection is through tandem gene duplication (Hahn 2009). Comparative genomics, aided by the emergence of high-quality genomes for many species, has uncovered the common role that tandem gene duplication plays in adaptive evolution to environmental challenges (Demuth and Hahn 2009; Hahn 2009; Lallemand et al. 2020; Brovkina et al. 2023). During tandem duplication, the number of copies of a gene can change expression levels or relax selection on both copies, potentially leading to functional diversification. Genome-wide, tandem duplications and resulting species-specific copy number variation are enriched for genes directly interacting with the biotic and abiotic environment rather than developmental or homeostatic genes (Demuth and Hahn 2009; Brovkina et al. 2023). Therefore, multi-component, protein-based molecular arms races between species may be fueled by tandem duplication, the characterization of which would enrich our understanding of how coevolution leads to functional divergence through genomic diversity.

Animal venom diversity is a prime example of adaptation through tandem gene duplication. Snake venoms are among the most well-understood venoms and are the product of several gene families that have undergone tandem duplication (Casewell et al. 2013; Dowell et al. 2016). Snake venom serine proteases (SVSPs) are one such protein family wherein genes have been duplicated several times, allowing for great diversity within and between species. From an ancestral duplication of the glandular kallikrein gene, SVSPs have diversified into gene copies with specificity for diverse targets, yielding proteins with potent pharmacological effects (Itoh et al. 1988). Venoms have a wide array of functions, many of which affect the physiology of blood clotting in envenomated species (Markland 1998; Serrano 2013).

Consistent with a coevolving system, many natural prey species are highly resistant to snake venom. Wild rodents, for example, are able to survive venom doses hundreds to thousands of times greater than that which would kill a lab mouse (*Mus musculus*) (Perez et al. 1978; Margres et al. 2017; Balchan et al. 2024). Despite the remarkable levels of resistance, we know very little about the molecular basis of resistance to snake venom proteases and the extent to which resistance and venom coevolve. Previously identified resistance factors (reviewed in Holding et al. 2016) largely include generic protease inhibitors such as α- and β-macroglobulins (de Wit and Weström 1987; Biardi et al. 2000; Omori-Satoh et al. 2000). α-2-macroglobulin, especially, is known to inhibit a broad spectrum of proteases, including serine proteases, metalloproteases, and cysteine proteases (Armstrong and Quigley 1999). This generalist activity allows α-2-macroglobulin to inhibit the diverse and variable protease contents of venom. Much less is known, however, about how protease inhibitors with more specific molecular mechanisms of inhibition may contribute to venom resistance.

A possible source of more specific inhibitors of snake venom is the serine protease inhibitor protein superfamily (SERPIN), members of which have previously been implicated in venom resistance (Barbour et al. 2002a; Gibbs et al. 2020). SERPINs share a fishing bait-like reactive center loop (RCL) that extends above the main globular body of the SERPIN and, in inhibitory SERPINs, mimics the target amino acid sequence of the protease(s) it inhibits (Gettins and Olson 2009). When a protease attempts to cleave the RCL of a SERPIN, the SERPIN will attempt to capture and inhibit the protease by forming an acyl intermediate that is then stabilized into a covalent bond resulting of loss of function for both the protease and the SERPIN (Lawrence et al. 1995; Gettins 2002; Huntington 2011). Alternatively, if hydrolysis of the acyl intermediate occurs before covalent bond formation, then the SERPIN is cleaved and rendered nonfunctional while the protease remains active.

Because of SERPINs' 1:1 inhibitory mechanism and the target-specific mimicry of the RCL, gene duplications of SERPINs may allow for mutational flexibility to target orthogonal proteases (Gettins 2002). Gene duplications have been found within the vertebrate-specific SERPINA subfamily of SERPINs, including α-1-antitrypsin (SERPINA1) and α-1-antichymotrypsin (SERPINA3) in rodents (Forsyth et al. 2003; Wu et al. 2023). Prior work with SERPINA1 paralogs in laboratory mice observed differential inhibition of several human proteases as well as variable patterns of inhibitory complex formation in the presence of snake venoms (Barbour et al. 2002a). A SERPINA1 paralog was also found to be enriched in tissue after envenomation in mice (Cavalcante et al. 2022). Beyond laboratory mice, a SERPINA1 paralog in ground squirrels (*Otospermophilus beecheyi*) has been found to be evolving at higher rates than other genes and is specifically retained when ground squirrel serum was passed over a venom-charged chromatography column (Gibbs et al. 2020; Ochoa et al. 2023). However, SERPINA1 is not the only SERPINA to see paralogous gene expansion in rodents. In the same tandem gene array, SERPINA3 is also heavily duplicated in mice and rats (Forsyth et al. 2003), compared to its single copy in the human genome. Together, these findings lead us to hypothesize that rodent SERPINA paralogs will include inhibitors of diverse SVSPs in snake predators.

Understanding if specific inhibitors contribute to venom resistance can better characterize prey's response to predators' diverse venom composition and offer insight into potential mechanisms of local adaptation, as gene-for-gene or similar molecular matching-based inhibition is the most likely to generate local adaptation and diversifying selection (Blanquart et al. 2013). Here, we combine comparative genomics, recombinant protein paralog expression, and direct functional tests to identify the rodent lineages in which SERPINA duplications occur most frequently and establish whether these duplications lead to functional variation in protease inhibition in a naturally-resistant wild rodent species, the Big-eared woodrat (*Neotoma macrotis*). Woodrats occur sympatrically with multiple rattlesnake species and some species of woodrats are known to be more than a thousand times more resistant to snake venom than laboratory mice (Perez et al. 1978 ). We specifically test the hypothesis that tandem duplication of SERPINA3-like genes of *N. macrotis* has produced inhibitors of serine proteases in the venom of its native rattlesnake predator *Crotalus oreganus* (Perez et al. 1978; de Wit 1982; Robinson et al. 2021).

## Results

### History of Gene Duplications in the SERPINA Locus

**Fig. 1.**
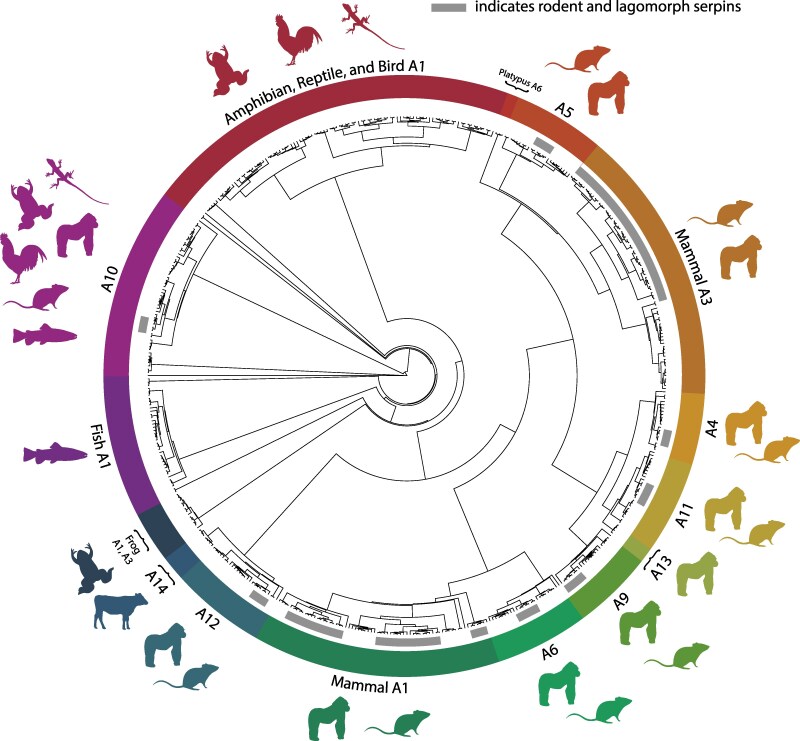
Evolution of SERPIN subfamily A genes at the tandemly-arrayed locus. Shown is a maximum-likelihood phylogenetic tree of 628 representative genes from 164 vertebrate species located at the SERPINA tandem gene array bounded by ppp4r4 and gsc genes. The colored segments of the circle represent different SERPINs, and the animal silhouettes adjacent to each segment show in which genome(s) that gene is present (amphibian, reptile, fish, bird, non-primate mammal, and primate). The gray inner arcs represent rodent and lagomorph paralogs of each gene, with the length of each arc being proportional to the number of duplications; there has been significant duplication of SERPINA1 and SERPINA3 compared to other animals, as well as other genes in the same tandem array. SERPINA10 was present in every class of animal represented in this study. Of note, SERPINA13 is unique to non-human apes and monkeys. Likewise, SERPINA14 is unique to non-rodent, non-simian mammals. Animal silhouettes were obtained from https://www.phylopic.org/.

Inferred evolutionary relationships of SERPINA paralogs within the conserved SERPINA tandem array (Fig. 1) make clear how the array differs in gene composition between species. Fish and non-mammal tetrapods have SERPINA1-like, A3-like, and A10-like genes, whereas mammals are unique in the presence of many single-copy SERPINA subfamily genes. Gene duplications are most frequent in SERPINA1-like and SERPINA3-like gene clusters in mammals (Table S1). Clades composed wholly of SERPINA1 and SERPINA10 contain representatives from each vertebrate class, suggesting that each serpin is derived from an ancestral vertebrate ortholog. The vast expansion seen in rodent and lagomorph copies of SERPINA1 and SERPINA3 implies selective pressure toward gene duplication.

**Fig. 2.**
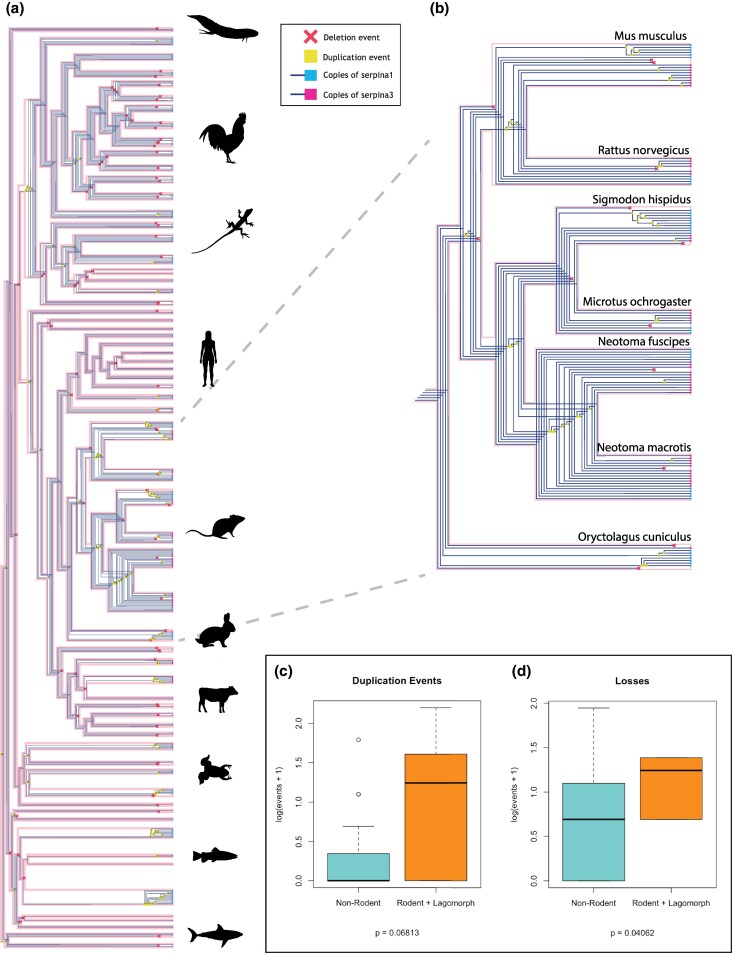
a) Reconciled gene tree created using GeneRax v2.1.3 and visualized using ThirdKind v3.5.0. The tree includes 203 SERPINA1-like and 80 SERPINA3-like sequences obtained from 66 vertebrate species. Duplications are represented by yellow squares, losses by red X's. The tips of the tree are colored to reflect the identity of the gene (SERPINA1 = cyan; SERPINA3 = pink). Duplications have independently occurred within mammals, fish, reptiles, and birds. b) The rodent clade of the reconciled gene tree. Most duplications occur within genera, many being repeated duplications of the same ancestral gene. c/d) Comparison of SERPIN c) duplication, and d) loss events in non-rodents vs. rodents and related lagomorphs. Rodents and lagomorphs have more frequent duplications (*P* = 0.068) and losses (0.041) than other species. A log scale was used to account for differences in variance between the two datasets.

From the reconciled gene tree and species tree (Fig. 2a and b), patterns of birth-death evolution in the gene family can be inferred. Rodents and related lagomorph species stand out in terms of copy number relative to other clades. Quantitatively, gene duplications and losses were on average higher in rodents and related lagomorphs than in other vertebrate species (*P* = 0.068; *P* = 0.041, respectively; Fig. 2c and d), indicating an overall elevated role of birth-death evolution in these lineages. Rodent genera experienced gene expansion to different extents and differed in final SERPINA1:SERPINA3 ratio. *Mus musculus* experienced four duplications each of SERPINA1 and SERPINA3, while *Rattus norvegicus* only had two duplications of SERPINA3 and none of SERPINA1. The four ancestral duplication events uniquely shared by these two species all occurred within SERPINA3s. New World rodents experienced three shared ancestral duplication events, all of which are sequential duplications stemming from SERPINA3. *Sigmodon hispidus* experienced one additional SERPINA3 duplication and six SERPINA1 duplications, while *Microtus ochrogaster* only experienced two additional SERPINA3 duplications. The two species of *Neotoma* share many duplication events in addition to those shared by New World rodents, likely reflecting their recent divergence, eventually resulting in the twelve total copies of SERPINA3. The most recent of the New World duplication events produced three of the *N. macrotis* SERPINA3 paralogs found to successfully inhibit proteases, as discussed below in greater detail.

### Signatures of Selection

**Fig. 3.**
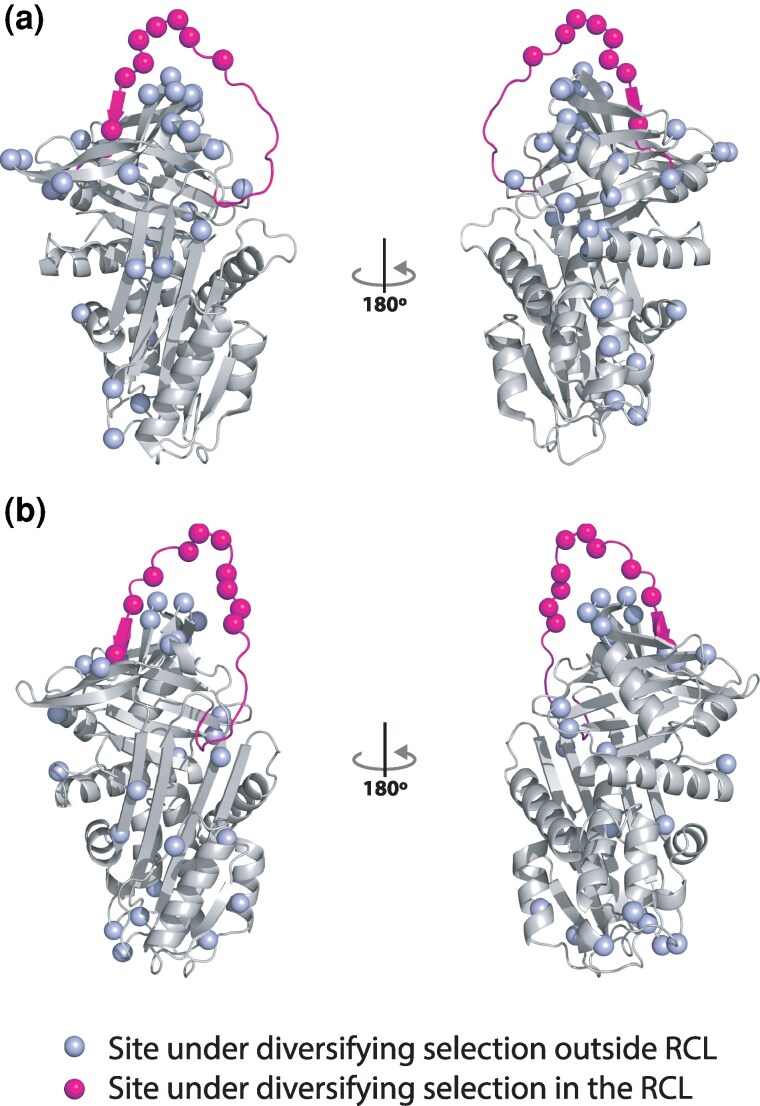
Sites experiencing episodic diversifying selection in SERPINA-1-like and SERPINA3-like genes of vertebrates. Sites under selection are indicated on the protein structurs with spheres; magenta spheres indicate sites within the RCL and pale blue spheres indicated sites in the main body of the SERPIN. Sites are mapped to the structures of a) human SERPINA1 (A1AT) and b) human SERPINA3 (A1ACT).

To investigate signatures of selection across the SERPINA1 and SERPINA3 gene sets, we implemented tests for recombination in GARD (Kosakovsky Pond et al. 2006), followed by a hierarchy of gene and site levels tests for selection. SERPINA1-like genes lacked evidence for recombination (GARD “no breakpoints” model ΔAIC_c_ = 3254.6), whereas SERPINA3-like genes showed evidence for break points at nucleotide positions 332 and 953 (GARD “2 breakpoint analysis” model ΔAIC_c_ = 35.3) and the resulting partitions were included in selection tests. We first tested for genewide evidence for episodic diversifying selection using BUSTED (Murrell et al. 2015), where both gene sets showed evidence for episodic diversifying selection in the duplicated rodent lineages relative to the broader vertebrate background (SERPINA1-like BUSTED *P*-value < 0.00001; SERPINA3-like *P*-value < 0.00001; Tables S2 and S3). Next, we tested individual sites for episodic diversification using MEME (Murrell et al. 2012), and found 48 such sites in SERPINA1-like genes and 37 sites in SERPINA3-like genes (*P* < 0.05; Fig. 3a and b; Tables S4–S5). In the SERPINA1-like and SERPINA3-like genes, 8 and 9 of the positively selected codons, respectively, were in the RCL. These were higher proportions than expected by chance given the total number of selected sites and total sequence length (SERPINA1-like: z = 2.9, *P* = 0.004; SERPINA3-like: z = 4.1, *P* < 0.0001), indicating that RCL are common targets of positive selection.

### Functional Diversification

**Fig. 4.**
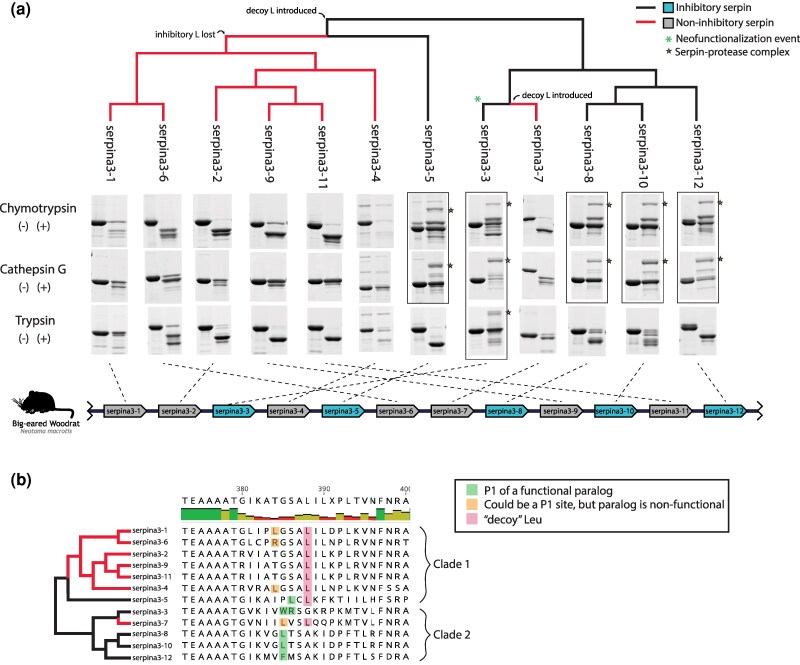
Evolution of SERPINA3-like gene function in *Neotoma macrotis.* a) Comparison of the function of woodrat SERPINA3 paralogs against chymotrypsin, cathepsin G, and trypsin, mapped onto a phylogeny of gene relationships of woodrat SERPINA3s. Two clades emerged, one that consisted primarily of paralogs that did not inhibit by any of the tested targets (subtended by red branches) and one that consisted primarily of inhibitory paralogs (subtended by black branches and enclosed in a black box. Most of the members of this inhibitory clade only inhibited chymotrypsin and cathepsin G, but SERPINA3-3 underwent a neofunctionalization event to inhibit trypsin (asterisk marking its evolutionary recency). SDS-PAGE images indicating inhibitory complex formation are boxed, and the corresponding genes on the genome are blue. In the left lane of each cropped gel result is the SERPIN-only control, whereas the right lane is the mixture of SERPIN and protease. High molecular weight SERPIN-protease complexes are marked with yellow star. Any intense bands uniquely in the protease-treated lane and below the molecular weight of the SERPIN-only control band reflect secondarily cleaved serpin. Protease-only controls and uncropped versions of each result can be found in Fig. S4 and independent replicate reactions are shown in Fig. S5. The genomic orientation of the genes encoding each paralog is indicated by the dotted lines; duplication events do not correspond with synteny on the chromosome. b) Comparison of the RCL region of all woodrat SERPINA3 paralogs in a multiple alignment with human SERPINA3. Known and suspected P1 residues are highlighted in green. SERPINA3-3 has consecutive Trp (W) and Arg (R) residues, which we hypothesize is the source of its neofunctionalization to inhibit trypsin as well as cathepsin G and chymotrypsin.

We successfully expressed all 12 SERPINA3 paralogs from *N. macrotis* with high purity, while only 2 out of 5 SERPINA1 paralogs were expressed, and with relatively low purity (Figs. S1 and S2). We therefore focused largely on SERPINA3 proteins for detailed functional characterization. SERPINA3 paralogs were reacted with cathepsin G, chymotrypsin, and trypsin. If inhibition occurs, we expect a protein band to form that is larger in molecular weight than either the purified SERPIN or the purified proteases, representing the inhibitory complex. Out of the SERPINA3s, paralogs 3-3, 3-5, 3-8, 3-10, and 3-12 formed complexes with both cathepsin G and chymotrypsin (Fig. 4a; Fig. S4). All of these, except SERPINA3-5, are in the same clade, henceforth “clade 2”; this clade also includes SERPINA3-7, which did not form complexes with either protease. SERPINA3-5 serves as the outgroup to the remaining SERPINs, henceforth “clade 1.” Along with variation in complex formation, SERPINA3 paralogs varied in the extent to which they were cleaved by the proteases. For example, almost all the SERPINA3-6 was cleaved by chymotrypsin, whereas other SERPINs resisted cleavage to some extent. However, SERPINA3-6 seemed to resist degradation past the initial cleavage, which was not common among other paralogs like SERPINA3-1 and SERPINA3-4, which were cleaved several times, forming multiple lower molecular weight bands.

Of the SERPINA3 paralogs that formed complexes with cathepsin G and chymotrypsin, the degradation patterns differed (Fig. 4a; Figs. S4 and S5). Most paralogs resisted degradation by cathepsin G much more effectively than they resisted degradation by chymotrypsin, despite a longer incubation time with the former. Only SERPINA3-7 was heavily degraded by cathepsin G. This likely reflects the more generalized substrate preferences of chymotrypsin compared to cathepsin G.

One SERPINA3 paralog (SERPINA3-3) formed an inhibitory complex with trypsin (Fig. 4a; Figs. S4 and S5). Like other proteases, trypsin shows differential primary and secondary cleavage patterns of SERPINA3 paralogs with most, but not all, SERPINs cleaved once but resisting further. There was no discernable difference in cleavage patterns between clades. Relative to the background of chymotrypsin-like protease inhibition in SERPINA3-like genes, trypsin-like protease inhibition of SERPINA3-3 is a significant neofunctionalization event that renders a broad inhibitory capacity in the resulting protein.

### Inhibition of SVSPs

**Fig. 5.**
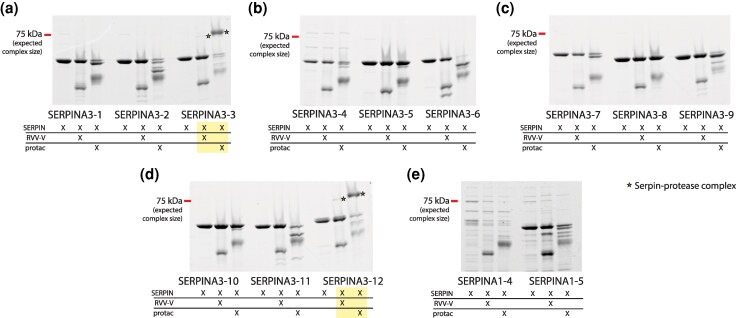
Inhibition of purified venom SVSP by SERPINA3-like proteins: each of the 12 SERPINA3 paralogs was tested against RVV-V activator and *A. contortrix* Protein C Activator (Protac) on Coomassie Blue-stained SDS-PAGE gels. SERPINA3-3 (highlighted) formed complexes with both SVSPs, but more readily reacted with Protac. Uncropped versions of these gels are provided in Fig. S6 and an independent replicate reaction is show in Fig. S7. The X marks below each lane specify the lane contents as either purified SERPIN or SERPIN + SVSP. High molecular weight SERPIN-protease complexes are marked with a yellow star, and conditions in these lanes are highlighted with yellow boxes. Any intense bands uniquely in the protease-treated lane and below the molecular weight of the SERPIN-only control band reflect secondarily cleaved SERPIN.

We next tested the SERPINA paralogs against two commercially available, purified SVSPs: RVV-V from the Russell's Viper (*Daboia russelli*) and Protac from the Copperhead (*Agkistrodon contortrix*). These SVSP paralogs have diverged into very specific activators of Coagulation factor V and protein C, respectively. Two woodrat SERPINA3 paralogs, SERPINA3-3 and SERPINA3-12, formed high molecular weight inhibitory complexes with these SVSPs (Fig. 5; Figs. S6 and S7). Both SERPINs formed relatively more complex with Protac than with RVV-V, suggesting some degree of target specificity among individual venom proteins.

Finally, we reacted the SERPINA3 paralogs with benzamidine-enriched SVSPs from natural rattlesnake predators of woodrats, *Crotalusoreganus*, *Crotalus molossus*, and *Crotalus adamanteus.* Complexes occurred between SERPINA3-3 and isolated SVSPs from all three snake species (Fig. 5; Figs. S8 and S9). The band containing complexes was thickest for the mixture of SERPINA3-3 and *C. adamanteus* SVSPs, and thinnest for the mixture with *C. oreganus* SVSPs. Three complexes of different molecular weights appeared in the mixture with *C. molossus*. All paralogs showed evidence for a substrate pathway, with a band appearing slightly under the molecular weight of the control SERPIN, suggesting that SVSPs can evade SERPIN cleavage (Figs. S8 and S9).

### Assessment of Function in the Presence of Glycosylation

One limitation of our work with *E. coli*-expressed SERPINs is the lack of glycosylation; many natural SERPINs expressed by eukaryotes are glycosylated, and some glycosylations have been shown to promote or inhibit SERPIN functional activity, depending on the type of terminal monosaccharide (Chernykh et al. 2023). To address this concern, we reexamined the inhibition patterns of the neofunctionalized and venom inhibiting paralog, SERPINA3-3, following expression in Chinese Hamster Ovary (CHO) cells. The purified protein appeared as a smear rather than discrete band, reflecting considerable and variable glycosylation, which was confirmed when enzymatic removal of N-linked glycans collapsed the smear back to a discrete band at the same size as that purified from *E. coli* (Fig. S10a). We incubated the CHO-expressed SERPINA3-3 with biotinylated proteases: trypsin, chymotrypsin, RVV-V, Protac, and the mixed SVSP fractions from the rattlesnakes *C. oreganus, C. molossus, and C. adamanteus*. We were unable to visualize complexes between the CHO-expressed SERPINA3-3 and biotinylated RVV-V, but secondary characterization of the biotinylated RVV-V showed that the addition of biotin ablated RVV-V's activity (data not shown). However, western blots to detect biotinylation did confirm high molecular weight SERPIN-protease complexes formed with trypsin, chymotrypsin, Protac, and the rattlesnake venom SVSPs, consistent with the functional results in the *E. coli* expressed form (Fig. S10b–d).

## Discussion

Even in laboratory mice, the specific roles of multiple SERPINA1 and SERPINA3 paralogs are poorly characterized (Forsyth et al. 2003). We have demonstrated that rodents have particularly high rates of SERPINA gene copy number evolution accompanied by evidence for positive selection. We have empirically demonstrated repeated functional diversification across these tandemly arrayed genes in a wild rodent system. We find support for our working hypothesis that at least some of these duplications serve as venom inhibitors, expanding the known functional repertoire of venom-inhibitory proteins to SERPINA3-like paralogs. Generally, rodent SERPINA3s show repeated gains and losses of inhibitory activity against chymotrypsin-like serine proteases. Additionally, we have identified one apparent neofunctionalization event toward the dual inhibition of trypsin- and chymotrypsin-like serine proteases, both of which may be conducive to coevolution with snake venom proteases that can be trypsin- or chymotrypsin-like at their substrate specificities (Whittington et al. 2018).

### History of Gene Duplications in the SERPINA Locus

In humans, SERPINA1 is one of the most abundant plasma proteins, where it exists as a single-copy gene whose product inhibits elastases (Gettins 2002). Similarly, SERPINA3 also exists as a single-copy gene in humans and primarily inhibits cathepsin G (Gettins 2002). Our analysis of gene gains and losses suggests that the large tandem arrays of SERPINA1 and SERPINA3 found in rodents developed via several independent gene duplication events, rather than stemming from one large ancestral duplication event, and that rodents and lagomorphs have evolved a tendency toward duplication in these genes relative to other vertebrate lineages. In addition, there is species-specificity in which gene is most common between SERPINA1 to SERPINA3, with the cotton rat (*Sigmodon hispidus*) having many more SERPINA1s than SERPINA3s, the woodrat and house mouse having more SERPINA3s than A1s, and the prairie vole and Norway rat having similar numbers. Our finding that most duplications occurred within rodent genera is consistent with previous studies that claim that duplications in mice primarily occurred at the shared ancestor of the genus *Mus* (Rheaume et al. 1994).

Our work raises comparative questions for future research in the study of phylogenetic and functional diversification of SERPINs. Our selection analyses indicated significant bouts of episodic diversifying selection in rodents relative to the broader vertebrate background, and that the RCL is a hotspot for positive selection. This finding places emphasis on the evolvability of the modular RCL, while calling for studies of the residues in the main body of the SERPIN that are also under selection. The SERPIN superfamily has an ancient origin and is thus shared across all domains of life, producing many diverged groups of SERPINs of varied function (Spence et al. 2021), including non-inhibitory roles such as carrier and signaling functions (Wang et al. 2014; Alvarez-Buylla et al. 2023). Functional annotation is still sporadic outside of human serpins, where there are signatures of coevolution with target proteases (Haynes et al. 2025). However, our current results call for analyses of the role different types of ecological or physiological demands (eg anti-parasites and anti-predators vs. homeostatic regulatory functions) play in the tendency of SERPINs to evolve by tandem duplication. In turn, tandem duplication has acted independently on several SERPINs, including the SERPINA subfamily studied herein, the SERPINB subfamily that is also found in vertebrates (Heit et al. 2013), the iripin SERPINs in ticks (Cerqueira de Araujo et al. 2025), and the PpSerpins from parasitoid wasps (Yang et al. 2024), which could facilitate comparative analyses of the means by which dynamic copy number evolution, adaptive radiation, and tandem genomic arrangement influence the exploration of the SERPIN sequence space (Spence et al. 2021).

### Functional Diversification

The RCL sequences of the SERPINs studied herein help explain the functional diversity we observed. Chymotrypsin preferentially cleaves after Trp (W), Tyr (Y), or Phe (F), with secondary affinity for cutting after Leu (L), Met (M), or His (H) (Keil 2012). Cathepsin G cleaves at similar sites to chymotrypsin (Thorpe et al. 2018), and it is therefore unsurprising that the same tested paralogs formed complexes with cathepsin G and chymotrypsin. However, SERPINs showed much greater resistance to primary and secondary cleavage by cathepsin G than they did to chymotrypsin, perhaps due to cathepsin G's more defined substrate specificity compared to that of chymotrypsin (Polanowska et al. 1998). Among the SERPINA3s tested in this study, SERPINA3-5, 3-8, and 3-10 have a Leu residue within one residue of the typical P1-P1′ region as observed on a multiple alignment with human SERPINA3 (Fig. 4b) (Horvath et al. 2005). Furthermore, SERPINA3-3 has a Trp residue in the same place, and SERPINA3-12 has a Phe residue.

Mapping of complex formation to the of *N. macrotis*'s SERPINA3 gene phylogeny showed a pattern suggesting that ancestral SERPINA3s likely had the ability to form a complex with and inhibit chymotrypsin, and that the ability was lost twice. The first of these two losses to occur was within clade 1, composed of SERPINA3-1, 3-2, 3-4, 3-5, 3-6, 3-9, and 3-11 SERPINA3-5 is the only member of clade 1 to have retained the ability to inhibit chymotrypsin. Clade 1 is characterized by the presence of a Leu residue in position 388 on the consensus sequence. Though Leu is a known cleavage site for chymotrypsin, Leu^388^ may be too far toward the C-terminal end of the RCL (Fig. S12) to facilitate the trapping action necessary for inhibition of a protease, therefore we term this residue as the “decoy” Leu (Fig. 4b). Only SERPINA3-5 was able to form a complex while also having a leucine residue at position 388. However, it also has another Leu residue at position 386, which we hypothesize serves as a kinetically favored cleavage target for chymotrypsin. Previous research has shown that certain SERPINA1 paralogs with seemingly valid P1-P1′ sites are unable to form a complex with chymotrypsin, likely due to structural differences outside of the RCL (Barbour et al. 2002a). The second loss of chymotrypsin inhibitory function occurred in clade 2. Most of the paralogs in this clade retain inhibitory function, but SERPINA3-7 has lost that ability. As stated before, there are several different functional residues at the P1 site in clade 2: Trp, Leu, and Phe, all at position 385 in the consensus sequence. While SERPINA3-7 maintains Leu^385^, it has also separately evolved the “decoy” Leu at position 388. This suggests that Leu^388^ can block inhibitory function, but does not preclude other possible mechanisms. These hypothesized links between sequence and functional variation are consistent with prior work (Riewald and Schleef 1996; Denardo et al. 2023; Wu et al. 2023) and underscore the readiness with which paralogous duplication can diversify function with only one or a few amino acid substitutions.

Neofunctionalization to a trypsin inhibitor in SERPINA3-3 is also explainable through minimal evolution of the RCL. Trypsin preferentially cleaves after positively charged lysine (Lys, K) and arginine (Arg, R) residues (Manea et al. 2007). In human SERPINA1, Met at P1 also successfully inhibits trypsin (Gettins and Olson 2009; Huntington 2011). Previous studies in mouse SERPINA1 paralogs have demonstrated even more freedom in the P1 site, with Tyr-Ser (S) residues at the scissile bond (Barbour et al. 2002a). Despite this apparent relative flexibility, in our study, only SERPINA3-3 formed a complex with trypsin, with Arg^386^ in a secondary P1 position (Fig. 4b). This indicates a neofunctionalization event where SERPINA3-3 uniquely evolved the capacity to inhibit trypsin while retaining the ability to inhibit chymotrypsin and chymotrypsin-like proteases. Generally, our results demonstrate how gene copies can lead to the evolution of altered protease inhibition profiles.

### Inhibition of SVSPs

Barbour et al. (2002a) showed that the five SERPINA1 paralogs in *Mus* are capable of SVSP inhibition, with each paralog forming unique patterns of inhibitory complexes depending on the snake species' venom composition. A major implication of our work is to extend these results beyond SERPINA1 paralogs to the SERPINA3 paralogs, which are often more gene copy-rich than SERPINA1. In addition, we examined venom resistance through the lens of adaptive inhibition of a wild rodent SERPINs and their natural predators and closely related, but allopatric, species.

**Fig. 6.**
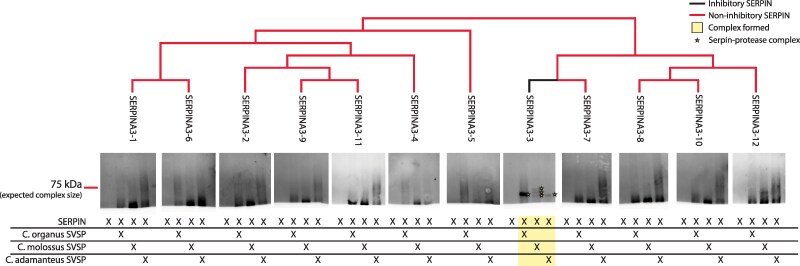
Complex formation with SVSP from rattlesnake predators. Western blots showing the potential of SERPINA3 paralogs to form inhibitory complexes with isolated SVSPs from three different rattlesnakes, ordered according to the phylogenetic relationships of woodrat SERPINA3 paralogs as indicated by the tree. The reaction took place in a 5:2 ratio of SERPIN to SVSP and at 37 °C. SERPINA3-3 formed a complex with all three snake SVSPs. These images depict the portion of each blot above 50 kDa for better exposure of the complexes; full versions of blot images are provided in Fig. S8, and select replicate reactions are show in Fig. S9. High molecular weight SERPIN-protease complexes are marked with a yellow star, and conditions in these lanes are highlighted with yellow boxes. Any intense bands uniquely in the protease-treated lane and below the molecular weight of the SERPIN -only control band reflect secondarily cleaved SERPIN.

Most of the snake species that overlap spatially with *N. macrotis* have primarily trypsin-like serine proteases (Whittington et al. 2018), as with all the venoms we tested herein. SERPINA3-3's neofunctionalization to a trypsin-like inhibitor may thus contribute to natural inhibition of these trypsin-like venoms (Fig. 6). Woodrat SERPINA3-3 formed complexes with SVSPs in all venoms tested, while the degree of complex formation varied. Different patterns of inhibition also occurred with the commercially available SVSPs RVV-V and Protac (Fig. 5), where woodrat's SERPINA3 paralogs were more effective inhibitors of Protac from *A. contortrix* than RVV-V from Russell's Viper. Russell's Viper is native to India and Bangladesh, while *A. contortrix* is native to Eastern North America (Wüster et al. 1992; Guiher and Burbrink 2008), suggesting potential adaptation to inhibit venoms from the clade of vipers (Western Hemisphere Crotalinae) with which woodrats have coevolved. Taken together, our results may reflect coevolutionary local adaptation of the snakes to avoiding woodrat inhibitors (since *C. oreganus* is the local predator of these *N. macrotis*), accompanied by a steep drop in inhibitory capacity of any kind when distantly related Eastern Hemisphere viper venom was used. Such patterns of differential susceptibility and adaptation across phylogenetic scales are consistent with findings for snake venom metalloproteinase inhibition by woodrats and squirrels (Pomento et al. 2016; Robinson et al. 2021), as well as patterns of host and non-host resistance in host-pathogen systems (Antonovics et al. 2013). However, these results remain speculative until quantitative measurement of interactions between many more SERPINs and purified proteases are conducted across scales of population and phylogenetic diversification, and until in vivo studies of SERPINA3-3's effects on venom lethality confirm its physiological relevance.

In conclusion, the results of this study show how frequent gene duplication can produce functional variation, especially in SERPINs, where the residues of the RCL near the scissile bond can have such a strong effect on the SERPIN's target specificity. Closest homology to human single-copy orthologs, especially homology of the entire sequence rather than the RCL, is therefore an unreliable indicator of function due to the one-to-many gene relationships between species and potential for rapid neofunctionalization via one or a few mutations. In addition, we have provided additional evidence that tandem duplication may play a role in coevolving systems, including predator venom toxicity and prey resistance. The systematic testing method we used can be applied to these and other tandemly arrayed genes to further elucidate functional relationships. SERPINA3 paralogs should be added to the growing list of resistance factors that protect mammals in molecular warfare with venomous snakes.

## Materials and Methods

### Genome Annotation

Chromosome-scale or large-scaffold-scale genomes were downloaded from NCBI's Genome resource (Sayers et al. 2022; Table S6). SERPINA protein sequences from UniProt (The UniProt Consortium 2023) were queried with tblastn (Altschul et al. 1990) with e-value of 10^−6^ against each genome to locate putative exons of SERPINA genes. A total of 66 mammal, reptile, bird, amphibian, and fish genomes were manually re-annotated at SERPINA tandem arrayed loci, and the resulting gene list was supplemented with the NCBI RefSeq annotations from another 98 species' genomes. We translated and annotated SERPINA coding sequences to establish gene order, microsynteny, and nomenclature using web blastp searches with default settings. For the putatively novel SERPINA genes in the genomes of the woodrats *Neotoma fuscipes* and *N. macrotis*, we further refined the coding sequence annotation using RNA-seq reads visualized with splice-aware alignment in HiSAT2 (Kim et al. 2019). We then named the paralogs of SERPINA1 and SERPINA3 according to their genomic order on the chromosome assembly (eg SERPINA3-1, SERPINA3-2, … SERPINA3-12). To assist the study of gene order relationships over evolutionary time, we applied similar nomenclature to our annotations of SERPINS in other species. In total, 628 genes were used for the phylogenetic analyses detailed below.

### Phylogenetic Analyses and Selection Tests

The coding sequences used to create the phylogeny were taken from the tandem array that, in humans, is on chromosome 14 between ppp4r4 and gsc. Other SERPINAs are present on additional chromosomes in humans and many other animals, but we chose to focus on this tandem array's evolution and function. To recover a species-tree aware phylogenetic reconstruction of the SERPINA gene tree, we generated a codon-alignment with MUSCLE (Edgar 2004) using the CLUSTALW weight scheme (Thompson et al. 1994) and kmer6_6 distance in the first iteration and pctid_kimura distance in the subsequent iterations (200 max iterations), then back-translating. Next, we used timetree.org (Kumar et al. 2022) to obtain a dated species-tree for the species present in our alignment. We then used GeneRax v2.1.3 (Morel et al. 2020) to produce a gene tree and reconciled it to the species tree with the model set to “UndatedDL” to infer duplication and loss events, and we used ThirdKind v3.5.0 (Penel et al. 2022) to visualize the reconciled trees. Finally, we compared the count of duplication and loss events of 203 SERPINA1-like and 80 SERPINA3-like genes from the 66 carefully curated manual annotations, contrasting clade Glires (Lagomorphs + Rodents) to the remaining 59 non-Glires vertebrate species using a two-sample *t*-test on the log-transformed count of events in each group.

We tested for selection separately on the SERPINA1-like and SERPINA3-like gene orthogroups from the above alignments using tools in HyPhy v.2.5.84 (Pond et al. 2005). Within each set, we tested for gene-wide episodic diversification in BUSTED (Murrell et al. 2015) using default settings, choosing genes from rodent lineages as foreground and those from other vertebrate lineages as background. Next, we tested for site-by-site evidence of episodic diversification using MEME (Murrell et al. 2012) using an α = 0.05 to determine significance. The total number of sites under selection in each orthogroup was used to determine the expected (null) frequency of sites under selection in the RCL region, and then a one sample proportion test was used to determine if the RCL was more enriched for positively selected sites than expected by chance. Site under positive selection were mapped to the structure 3NE4 for SERPINA1/A1AT (Patschull et al. 2011) and the AlphaFold2 (Jumper et al. 2021) structure AF-P01011-F1 for SERPINA3/A1ACT provided by Uniprot (The UniProt Consortium 2023).

### SERPIN Expression and Purification

Each gene of interest was cloned into a pET-24(+) plasmid vector between the BamHI and XhoI sites (Fig. S1), with a Strep tag at the N-terminus, as well as a FLAG tag followed by a His_6_ tag at the C-terminus. Signal peptides were identified using signalP5 (Almagro Armenteros et al. 2019) and not included in the cloned sequence. No signal peptide was detected for either SERPINA3-5 and SERPINA3-6. Each tag was separated from other tags or the gene sequence by a Gly-Ser-Ser-Gly flexible linker. Constructs were synthesized at Twist Bioscience (South San Francisco, CA, USA) and full constructs are included in Table S7. These vectors were chemically transformed into NiCo21(DE3) Competent *E. coli* (New England BioLabs Inc., Ipswich, MA, USA; #C2529H) and selected with 30 ug/mL kanamycin.

*E. coli* were cultured in LB broth at 37 °C to an optical density of 0.6 to 0.9 abs at 600 nm, followed by 0.4 mM IPTG to induce expression before further incubation at 37 °C for 2 hours. Cells were recovered by centrifugation at 6,000*×g* for 20 min at 4 °C, washed with 0.85% NaCl, pelleted again, and resuspended in 1 mL HBS (25 mM HEPES, 150 mM NaCl, pH 7.4) for every 100 mL of culture. To prepare lysate, 70 uM lysozyme, 110 nM DNaseI, 5 mM MgCl2, and 0.5 mM CaCl_2_ were added and incubated while rotating for 30 min at room temperature. Three freeze-thaw cycles were performed with liquid nitrogen, then the lysate was cleared by centrifugation at 16,000*×g* for 20 min at 4 °C, passed through a 0.22 μm filter, and diluted to 1:4 in HBS containing 5 mM imidazole (BioUltra, ≥99.5%; Sigma, Burlington, MA, USA; #56749-250G).

SERPIN paralogs were purified using fast protein liquid chromatography (FPLC) on an AKTÄ Pure FPLC system (Cytiva Corp., MA, USA) with a HiTrap TALON crude 1 mL column (Cytiva Corp.) equilibrated with HBS containing 5 mM imidazole. Lysate was then applied and washed with HBS containing 7.5 mm imidazole until the UV measurements were stable (within 0.1 mAU) for 1 min. SERPIN paralogs were eluted in HBS containing 150 mM imidazole and buffer exchanged into HBS using a HiPrep™ 26/10 Desalting column. Protein containing fractions were pooled, aliquoted, and stored at −80 °C.

Purity was assessed by electrophoresis using Invitrogen Novex Wedgewell™ 4 to 20% Tris-Glycine polyacrylamide gels (Waltham, MA, USA; XP04205) under reducing conditions, loading 1.2 ug of total protein into each well and staining with Invitrogen SimplyBlue SafeStain. To differentiate target proteins from low-abundance impurities, a western blot was performed, using the previously stained gels. A three-step dry transfer process was used, beginning at 20 V for 1.5 min, then 23 V for 6 min, and finally 25 V for 3 min (iBlot 2, Invitrogen). The membranes were blocked with 2% bovine serum albumin (BSA) (Sigma-Aldrich; #A7906) in Tris-buffered saline plus Tween (TBS-T) (20 mM Tris, 500 mM NaCl, pH 7.5; with 0.1% Tween-20). Western blots were probed with a primary mouse anti-Strep-II tag antibody (Abcam, Inc; Cambridge, UK, AB184224, Lot #1033268-2) at a 1:1000 dilution in TBS-T for two hours, followed by a secondary goat anti-mouse horseradish peroxidase (HRP)-conjugated antibody (1:5000 dilution) for one hour (BioRad, #170-6516). Three fifteen-minute washes in TBS-T were conducted between antibody applications and before addition of the chemiluminescent substrate (SuperSignal™ West Femto Maximum Sensitivity Substrate, Thermo Scientific, #34095). The chemiluminescent substrate was applied for 3 min before imaging by a ChemiDoc™ MP (BioRad) at auto-optimal exposure. For the A3 paralogs, 1 ug was loaded and for the A1 paralogs, 1.2 ug was loaded.

SERPINA3-3 was additionally expressed in CHO cells using the TurboCHO^TM^ expression system by Genscript Biotech (Piscataway, NJ, USA) with the manufacturer's standard protocols. The recombinant SERPINA3-3 construct includes GenScript Biotech's preferred signal peptide followed by N-terminal FLAG tag, and a C-terminal 6xHIS tag, the latter of which was used for purification from conditioned media (Table S8). We assessed the purity, migration profile, and glycosylation state of CHO-expressed SERPINA3-3 by SDS-PAGE under reducing conditions on an Invitrogen Novex Wedgewell™ 4-20% Tris-Glycine gel, comparing 1ug of untreated product with the same following deglycosylation treatment with 500 units (1 uL) of PNGase F (New England Biolabs, Ipswich, MA, USA) overnight at 37 °C.

### Enrichment and Biotinylation of SVSPs

Lyophilized whole venom was diluted to 20 mg/mL in HBS and applied to a HiTrap Benzamidine FF column (Cytiva Corp.) equilibrated with 0.05 M Tris-HCl containing 0.5 M NaCl (pH 7.4). Fractions of 0.5 mL were eluted with 0.05 M glycine (pH 3.0) and neutralized with 7.5 uL 1 M Tris-HCl (pH 9). Fractions with concentration of >0.150 mg/mL were pooled and buffer-exchanged into 25 mM HEPES containing 150 mM NaCl (pH 7.4).

Enriched SVSP fractions were biotinylated for sensitive detection in western blots of the serine protease mixture. Biotin was attached to SVSPs using the EZ-Link Micro Sulfo-NHS-Biotinylation Kit (Thermo Scientific, MA, USA; #21925) with a 20 mM fold-excess of biotin and approximately 1 mg of input venom protein. Biotinylated venom was buffer exchanged and recovered using a 5 mL Zeba Spin Desalting Column (Thermo Scientific; #89891), aliquoted, and stored at −80 °C until used. Detection of biotinylated venoms was achieved through western blot as described above, but using a single step incubation with HRP-conjugated streptavidin diluted 1:7500 in TBS-T, followed by three fifteen-minute washes in TBS-T.

### Reactions of SERPINs and Proteases

Functional variation was assessed by visualizing inhibition of the proteases cathepsin G, chymotrypsin, trypsin, and elastase, the human substrates of human SERPINA1 and SERPINA3. These proteases are the known targets of human SERPINA1 and SERPINA3, and are used here as reporters in the absence of identified rodent-specific targets. Proteases and SERPINs were mixed at a 2:5 molar ratio, respectively (0.9 μM protease and 2.25 μM SERPIN). Proteins were incubated at 22 °C on an Eppendorf Thermomixer R at 300 rpm. Empirically determined incubation times were as follows: SERPIN-chymotrypsin mixtures were incubated for 2 min, the SERPIN-trypsin mixtures were incubated for 1 min, the SERPIN-elastase mixtures were incubated for 5 min, and the SERPIN-cathepsin G mixtures were incubated for 5 min. Mixtures were quenched with 4X Laemmli Blue buffer (BioRad; #1610747) containing 10% 2-Beta-mercaptoethanol as a reducing agent and immediately boiled at 95 °C for 10 min before SDS-PAGE.

For mixtures of SERPINs and benzamidine-purified SVSPs, venom proteases and SERPINA3 paralogs were again mixed at a 2:5 ratio, respectively, and incubated at 37 °C for 20 min. As before, reactions were quenched and boiled. Gel samples were run on Invitrogen Novex™ Wedgewell™ 10% Tris-Glycine Plus polyacrylamide gels (#XP00105). The biotinylated venom and Strep-II tag-labeled SERPIN were visualized simultaneously with a western blot. The membranes were blocked in 2% BSA in TBS-T (0.01% Tween-20) overnight, then probed with 1:7500 N100, HRP-conjugated streptavidin and developed with chemiluminescent substrate (SuperSignal™ West Femto Maximum Sensitivity Substrate). To optimize the imaging process for detecting the complexes of interest in the presence of greater amounts of unbound protein, the portion of each blot above the 50 kDa marker was imaged separately at a longer exposure.

In addition to the benzamidine-purified SVSPs, the activity of SERPINA paralogs was tested against two commercially purified SVSPs, Russell's viper venom factor V activator (RVV-V) (Haematologic Technologies, Inc., Essex Junction, VT, USA; #RVVV-2000) and *A. contortrix* Protein C Activator (Protac) (Enzyme Research Laboratories, South Bend, IN, USA; #113-01). SERPINA paralogs were mixed with these SVSPs at a 1:1 molar ratio and incubated at 37 °C for 20 min then quenched, boiled, run on 4-20% Tris-Glycine polyacrylamide gels, stained with Invitrogen SimplyBlue™ SafeStain, and imaged.

## Supplementary Material

msaf290_Supplementary_Data

## Data Availability

The data for these experiments are all available in the article and online supplemental information.
