# Supplementary material for: Tandem Duplication of Serpin Genes Yields Functional Variation and Snake Venom Inhibitors
Source: Mol Biol Evol. 2025 Nov 11;42(11):msaf290. doi: 10.1093/molbev/msaf290 (PMC12646145; doi:10.1093/molbev/msaf290)
Supplement: msaf290_Supplementary_Data [file msaf290_supplementary_data.zip › Supplemental_Figures_.pdf]

Supplemental Figures

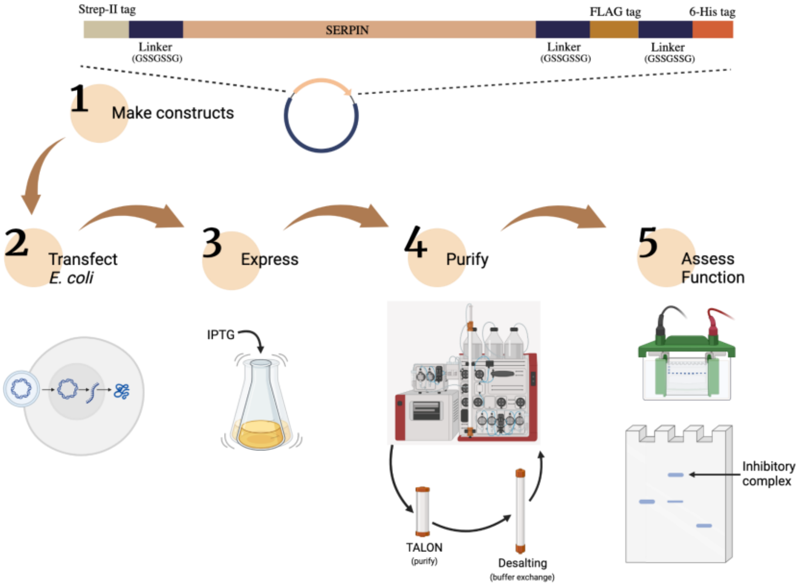

Figure S1. Overview of our bacterial expression, purification, and functional study of SERPINA constructs. Constructs were created from all paralogs of SERPINA1 (n=5) and SERPINA3 (n=12) in *Neotoma macrotis*, the big-eared woodrat. Created using BioRender.com.

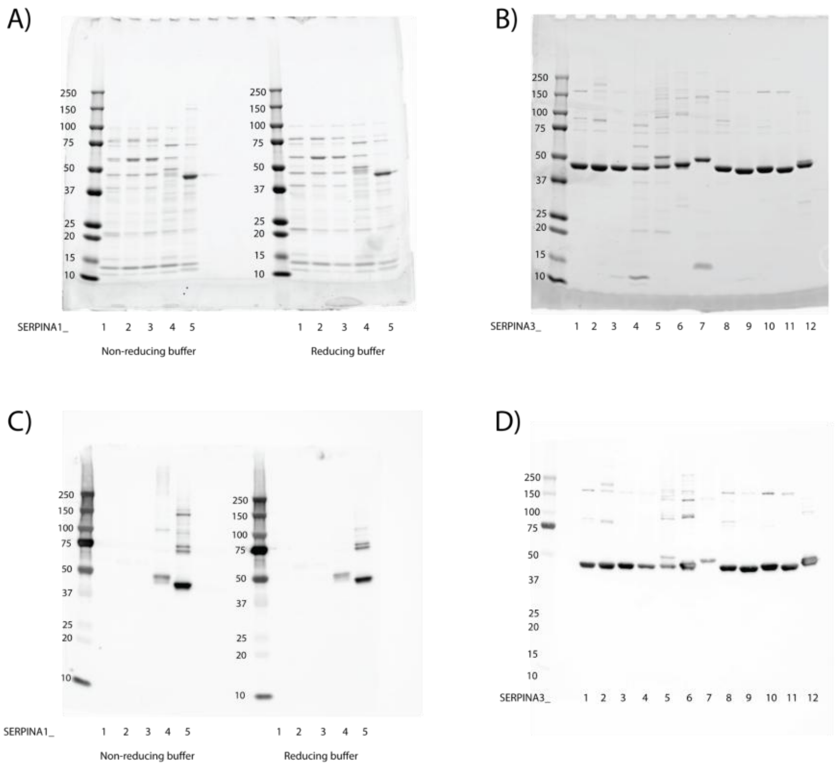

Figure S2. Purity analysis of expressed SERPINA1s (a, c) and SERPINA3s (b, d) of *N. macrotis*. Coomassie gels showed low purity in SERPINA1 paralogs (a) but high purity for SERPINA3 paralogs (b). The low purity of SERPINA1 paralogs cannot be attributed to disulfide bonding between SERPINs, as the non-reducing and reducing lanes look almost identical. SERPINA3s were only run with a non-reducing buffer, and most of the impurities appear to be disulfide-bonded SERPINs. Western blots using the N-terminal Strep-II tag showed that SERPINs A1-1, 1-2, and 1-3, were not successfully expressed, and only paralogs 1-4 and 1-5 were present (c). Meanwhile, all SERPINA3s were successfully expressed (d).

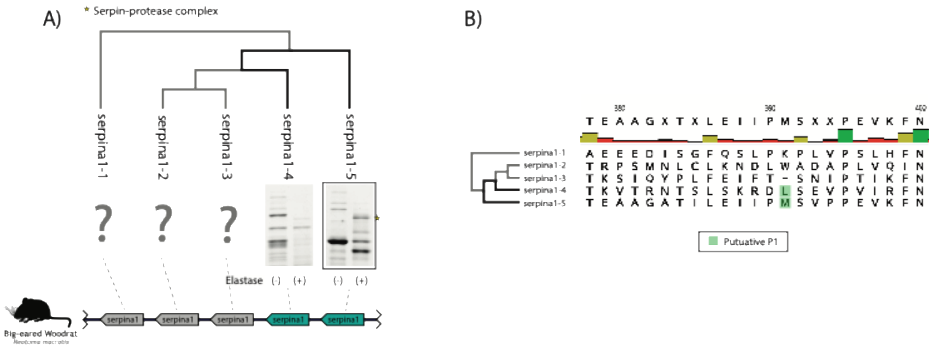

Figure S3. (a) Comparison of the function of SERPINA1-4 and 1-5 against elastase, mapped onto a phylogenetic tree of woodrat SERPINA1s. In the left lane of each cropped gel result is the SERPIN-only control, whereas the right lane is the mixture of SERPIN and protease. Protease-only controls and uncropped versions of each result can be found in Figure S4. Both paralogs formed a complex with the elastase at approximately the same molecular weight, with SERPINA1-5's complex being much darker. The location of the genes encoding each paralog is indicated by the dotted lines; all duplication events resulted in adjacent genes. (b) Comparison of the RCL region of all woodrat SERPINA1 paralogs. Both paralogs' P1 residue is traditionally capable of facilitating cleavage by elastase, with SERPINA1-5's M-S P1-P1' matching that of human SERPINA1. Evolutionary contextualization of SERPINA1 function is difficult without evidence for or against inhibitory ability for the majority of the SERPINA1 paralogs. Upon examining the RCLs of the unrepresented paralogs, few putative cleavage sites for elastase, trypsin, chymotrypsin, or cathepsin G were found.

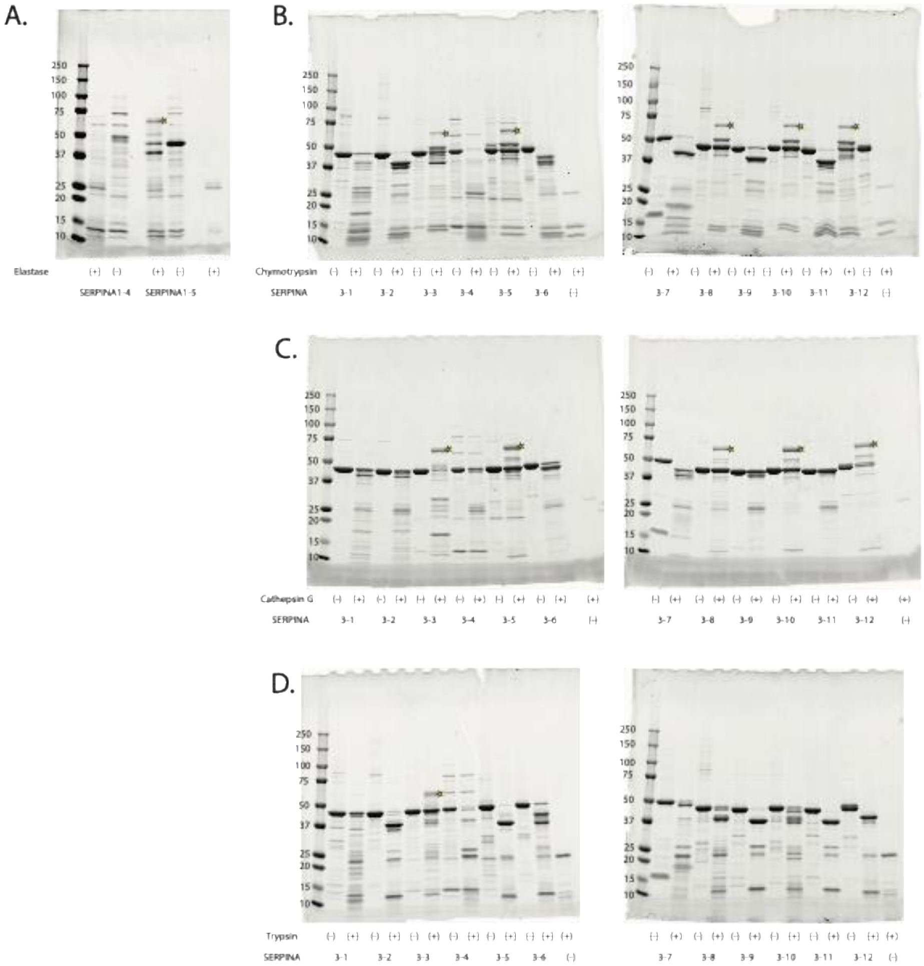

Figure S4. Uncropped versions of the Coomassie-stained gels (Fig. 4a) for 2:5 mixtures of neutrophil elastase (a), chymotrypsin (b), cathepsin G (c), and trypsin (d) with SERPINA paralogs. Paralog number is indicated across the bottom, as well as presence or absence of protease. High molecular weight SERPIN-protease complexes are marked with a yellow star, and conditions in these lanes are highlighted with yellow boxes. Any intense bands uniquely in the protease-treated lane and below the molecular weight of the SERPIN-only control band reflect secondarily cleaved SERPIN.

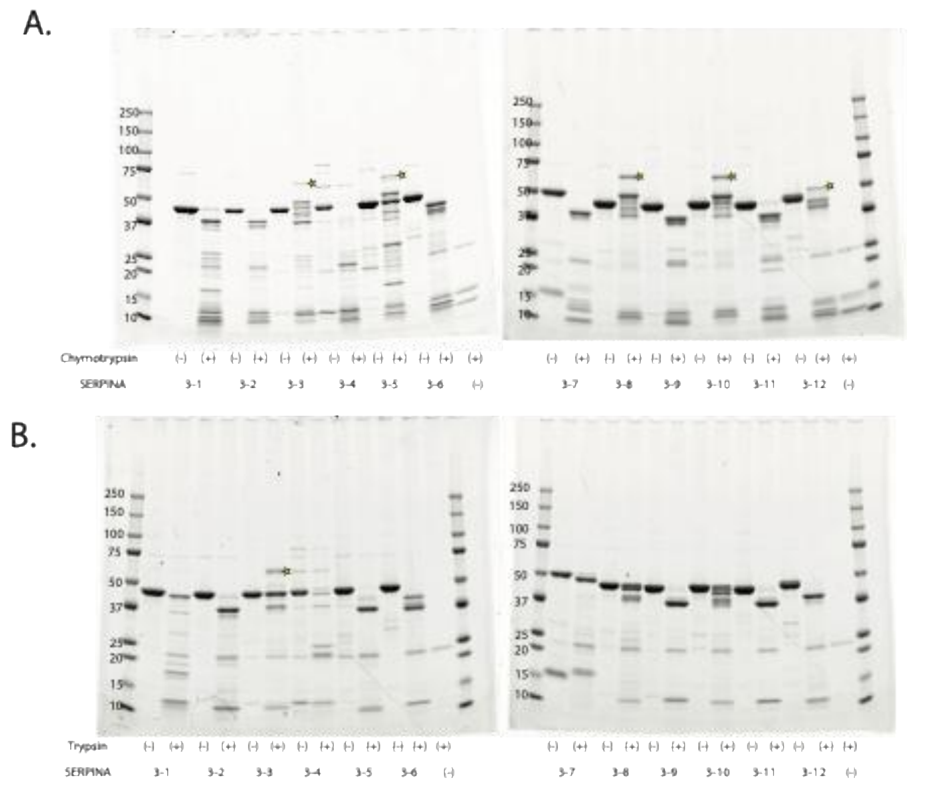

Figure S5. Coomassie-stained gels showing duplicate reactions for secondary confirmation of the results in Fig. 4a and Fig S4. Reactions were for 2:5 mixtures of chymotrypsin (a), or trypsin (b) with SERPINA paralogs. Paralog number is indicated across the bottom, as well as presence or absence of protease. High molecular weight SERPIN-protease complexes are marked with a yellow star, and conditions in these lanes are highlighted with yellow boxes. Any intense bands uniquely in the protease-treated lane and below the molecular weight of the SERPIN-only control band reflect secondarily cleaved SERPIN.

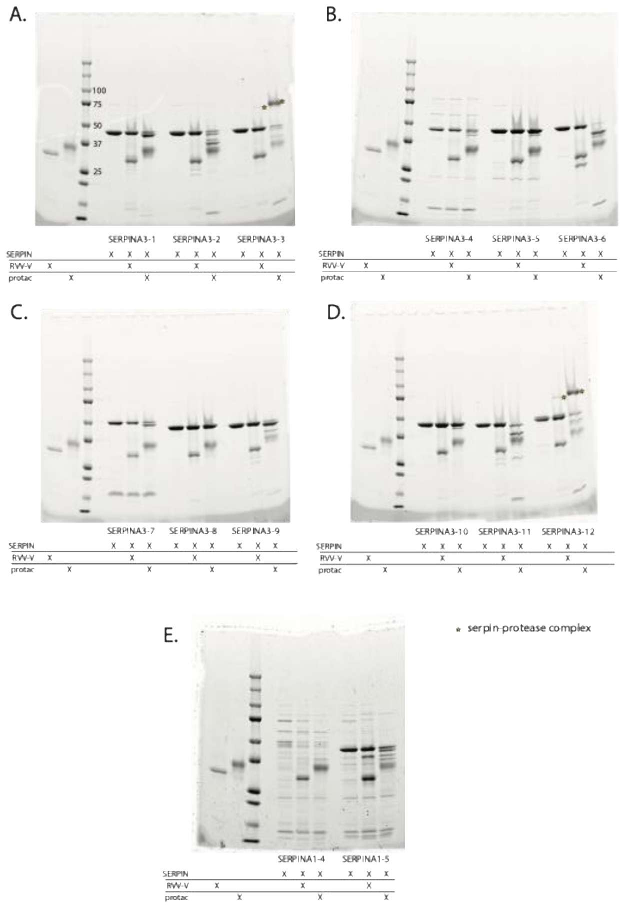

Figure S6. Uncropped versions of the Coomassie-stained gels (Fig. 5A-E) for 2:5 mixtures of Russell's viper venom FV activator (RVV-V) and copperhead venom protein C activator (Protac) with SERPINA paralogs. Paralog number is indicated across the bottom, as well as presence or absence of protease. High molecular weight SERPIN-protease complexes are marked with a yellow star, and conditions in these lanes are highlighted with yellow boxes. Any intense bands uniquely in the protease-treated lane and below the molecular weight of the SERPIN-only control band reflect secondarily cleaved SERPIN.

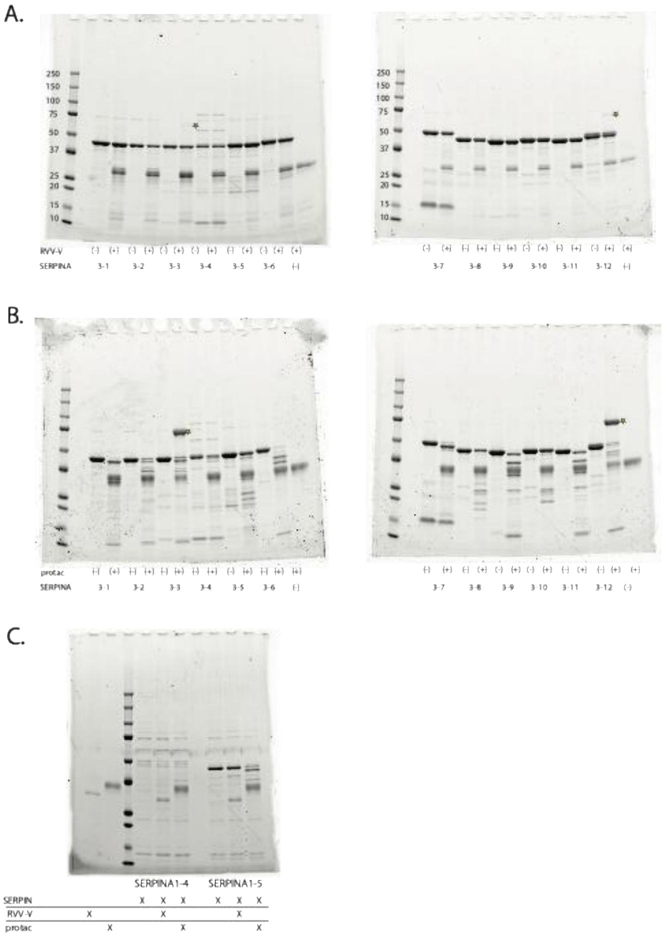

Figure S7 Coomassie-stained gels showing duplicate reactions for secondary confirmation of the results in Fig. 5 and Fig S8. Reactions were for 2:5 mixtures of Russell's viper venom FV activator (RVV-V) and copperhead venom protein C activator (Protac) with SERPINA paralogs. Unlike in Fig. S8, RVV-V and protac reactions with SERPINA3-like proteins were visualized on separate gels. Paralog number is indicated across the bottom, as well as presence or absence

of protease. High molecular weight SERPIN-protease complexes are marked with a yellow star, and conditions in these lanes are highlighted with yellow boxes. Any intense bands uniquely in the protease-treated lane and below the molecular weight of the SERPIN-only control band reflect secondarily cleaved SERPIN.

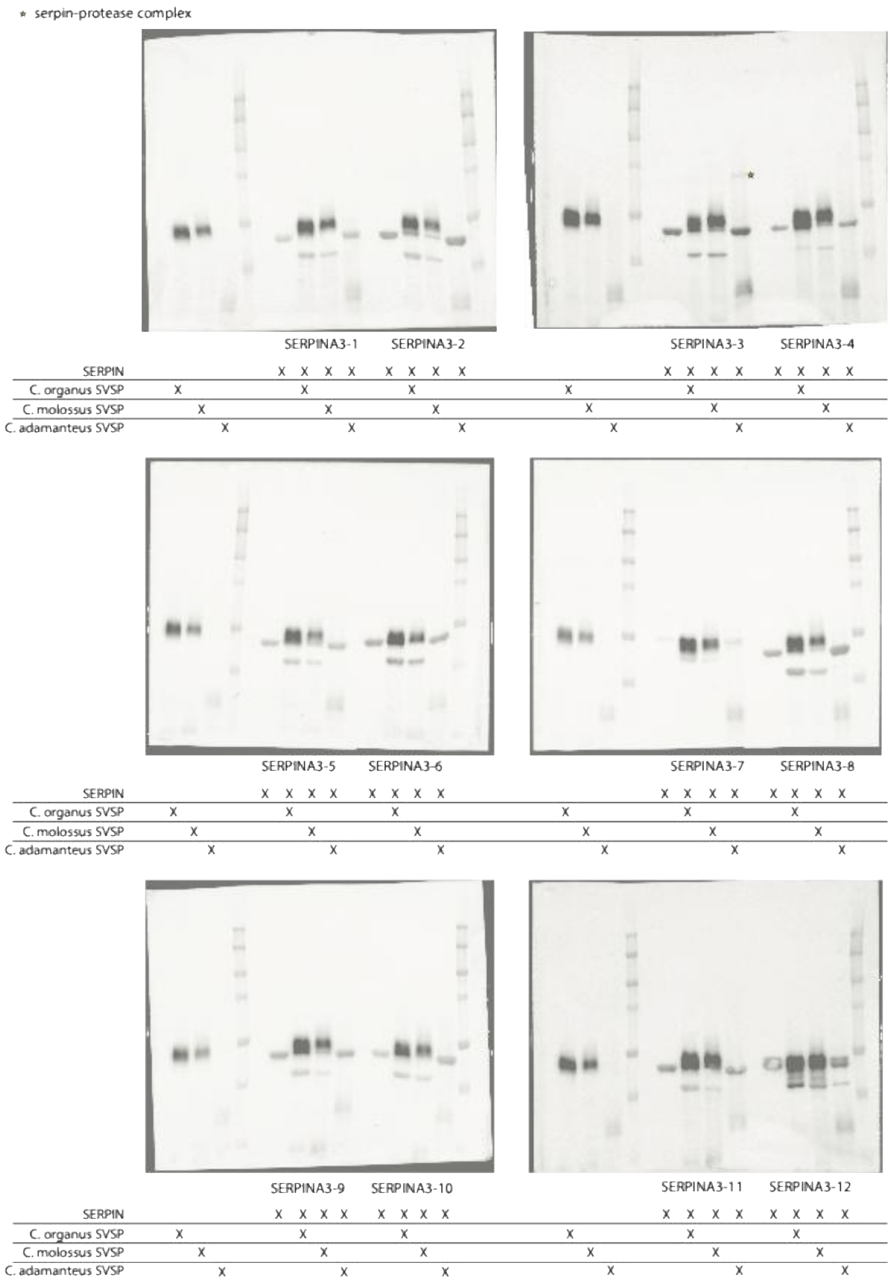
* serpin-protease complex
SERPINA3-1
SERPINA3-2
SERPINA3-3
SERPINA3-4
SERPINA3-5
SERPINA3-6
SERPINA3-7
SERPINA3-8
SERPINA3-9
SERPINA3-10
SERPINA3-11
SERPINA3-12
SERPIN
C. organus SVSP
C. molossus SVSP
C. adamanteus SVSP

Figure S8. Uncropped versions of SERPIN/benzamidine-purified SVSP blots from Figure 6. Blots were developed with HRP-conjugated streptavidin. Paralog number is indicated across the bottom, as well as presence or absence of protease. Visbile high molecular weight SERPIN-protease complexe bands are marked with a yellow star, and conditions in these lanes are highlighted with yellow boxes. Any intense bands uniquely in the protease-treated lane and below the molecular weight of the SERPIN-only control band reflect secondarily cleaved SERPIN.

Commented [HL1]: label molecular weights

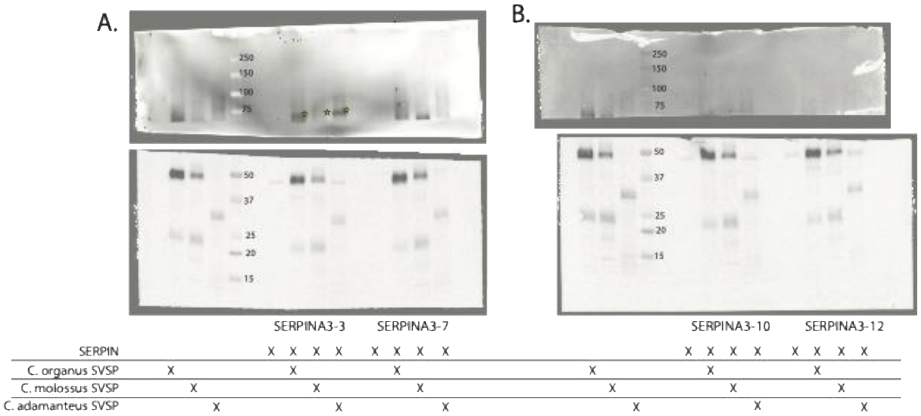

Figure S9 Replicate reactions of select SERPINA3 paralogs with SERPIN/benzamidine-purified SVSP blots from Figure 5. Unlike in Fig. 5/Fig. S6, gels were cut just below the 75kDA marker prior to transfer to the blotting member to develop independent blots of the larger and smaller protein bands. Panel A shows replicated SERPINA3-3 (positive hit for a SERPIN/SVSP complex in Fig6) and its sister gene SERPINA3-7. Panel B shows SERPINA3-10 and its sister gene SERPINA3-12. Blots were developed with HRP-conjugated streptavidin. Paralog number is indicated across the bottom, as well as presence or absence of protease. Visible high molecular weight SERPIN-protease complex bands are marked with a yellow star, and conditions in these lanes are highlighted with yellow boxes. Any intense bands uniquely in the protease-treated lane and below the molecular weight of the SERPIN-only control band reflect secondarily cleaved SERPIN.

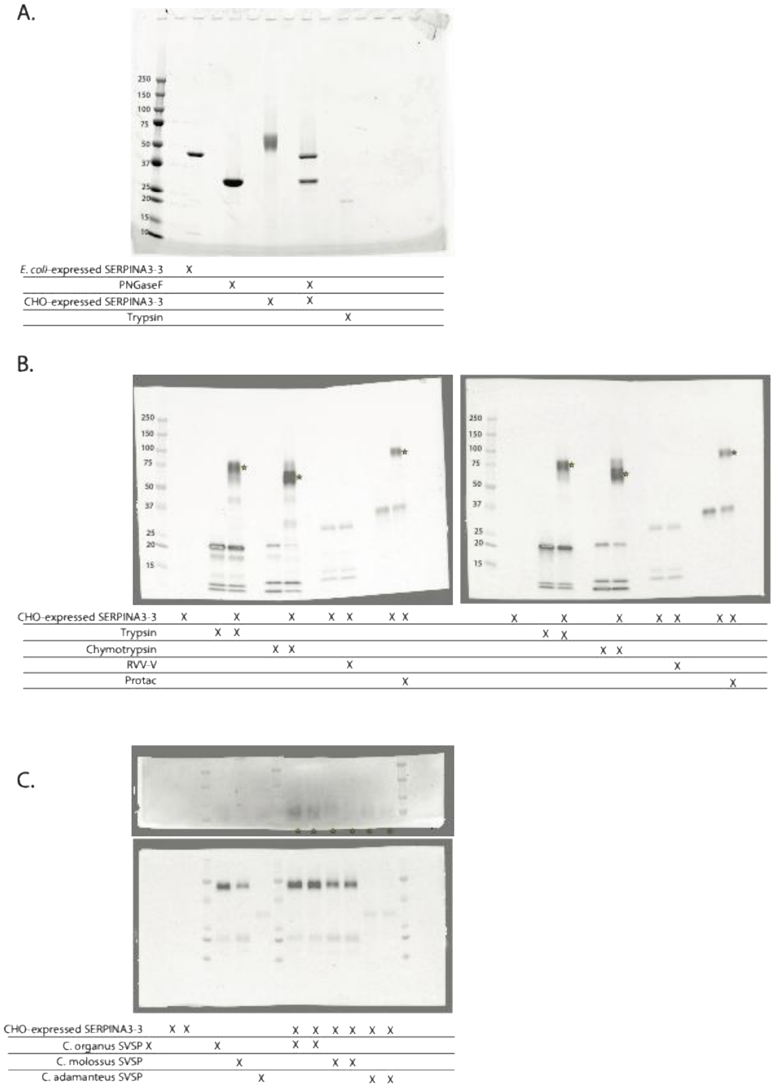

Figure S10 Investigation of size and activity of CHO-cell expressed, glycosylated form of SERPINA3-3 from *Neotoma macrotis*. A) Coomassie-stained SDS-PAGE gel run under reducing conditions contrasting *E. coli*-expressed and CHO-cell expressed SERPINA3-3 protein, and including N-glycosidase treatment with PNGaseF. B) Western blots of replicate reactions of CHO-cell expressed SERPINA3-3 with biotinylated proteases trypsin, chymotrypsin, RVV-V, and Protac. High molecular weight SERPIN-protease complexes are indicated with a yellow star. C) Western blot of replicate reactions of benzamidine purified SVSPs from wild

rattlesnake venoms with CHO-cell-expressed SERPINA3-3. Blots were treated with HRP-conjugated streptavidin followed by chemiluminescent imaging to detect biotinylated proteases.

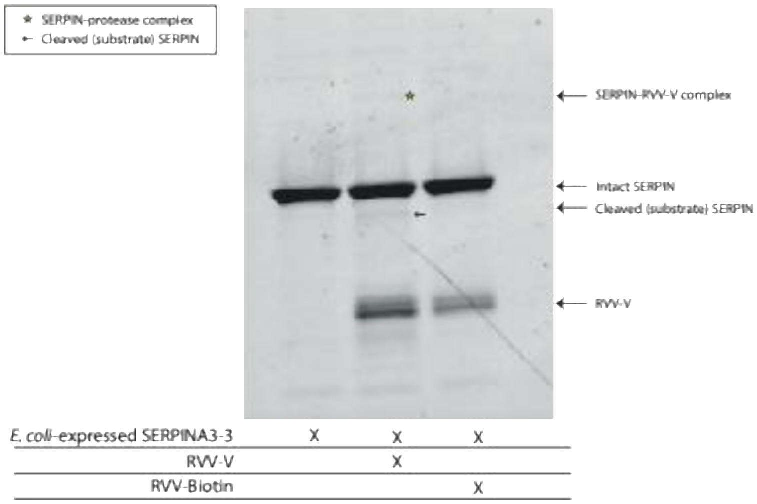

Figure S11. Biotinylation resulted in a loss of activity of Russell's Viper Venom FV activator (RVV-V). Coomassie staining of reducing SDS-PAGE gel, where *E. coli* expressed SERPINA3-3 was reacted with native and biotinylated Russell's viper venom FV activator (RVV-V). High molecular weight SERPIN-protease complex is indicated with a yellow star, small arrow in second lane indicates cleaved (substrate pathway) SERPIN, and the identities of various bands are further indicated to the right of the image.

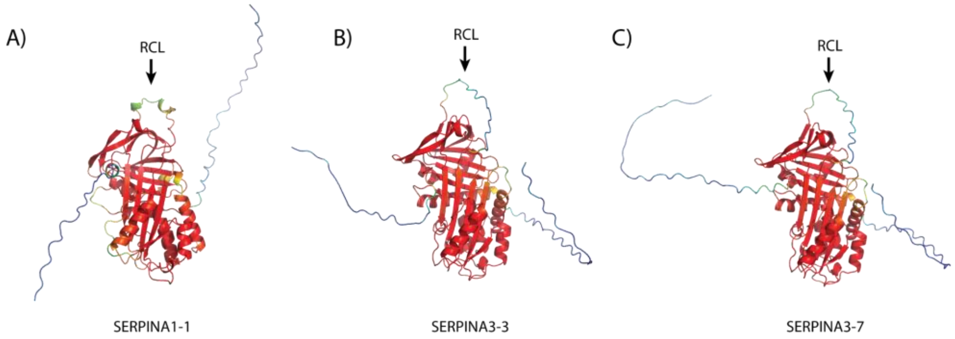

Figure S12. Alphafold2 structures of 3 SERPINA paralogs of interest: (a) SERPINA1-1, which was not successfully expressed and has an alpha-helix predicted in the RCL; (b) SERPINA3-3, which neofunctionalized to inhibit trypsin as well as chymotrypsin and cathepsin G; and (c) SERPINA3-7, SERPINA3-3's closest relative in the woodrat, which lost inhibitory function against all proteases tested in this study. Note that these structures include the N-terminal and C-terminal tags, which are not present in the natural sequence of these SERPINs and thus appear as low-confidence predictions on either side of the protein. SERPINA1-1, a more distant relative to other mammal SERPINA1s, contains a Lys residue at position 391. However, this Lys is flanked by two Pro residues, which is an extremely atypical pattern for an RCL; upon simulating the structure of SERPINA1-1, an alpha-helix was predicted in the RCL, which could render that paralog non-functional as an inhibitor through the traditional SERPIN mechanism.
